# Untangling the biochemical warfare of plant defence mechanisms against *Meloidogyne* spp.: a global systematic review with Sub-Saharan Africa insights

**DOI:** 10.1007/s00425-026-05035-6

**Published:** 2026-05-31

**Authors:** Ndivhuwo Ramatsitsi, Alen Manyevere

**Affiliations:** Department of Agronomy, University of Fort Hare, Private Bag X1314, Alice, 5700 South Africa

**Keywords:** Interactions, Resistance, Root-knot nematode, Susceptible, Tolerant

## Abstract

**Main conclusion:**

*Meloidogyne* spp. threatens diverse crops, but integrating biochemical resistance markers with agroecological practices and enhanced SSA research capacity is crucial for sustainable nematode management.

**Abstract:**

Root-knot nematodes (*Meloidogyne* spp.) pose a major threat to global agriculture, especially in Sub-Saharan Africa (SSA), where smallholder farming systems predominate. Their impact is frequently underestimated due to difficulties in diagnostic challenges and symptom overlap with other stresses. This systematic review critically examines 30 experimental studies conducted across 17 countries, 25 from other regions and 5 from SSA, examining plant biochemical defence responses against *Meloidogyne* spp., with an emphasis on SSA while incorporating global data. Literature was sourced from PubMed, Web of Science, Scopus, and AGRIS using strict inclusion criteria. Findings show that among the SSA studies included in this review, enzymatic assays were commonly employed on staple crops like maize, yam, and tomato in controlled environments, whereas global studies apply chromatographic metabolite profiling and molecular or omics-based analyses. Resistance and tolerance were consistently linked to elevated peroxidase (POD) and phenylalanine ammonia-lyase (PAL) activity, phenolic accumulation, and activation of key phytohormone pathways (salicylic acid, jasmonic acid, and ethylene). However, major limitations identified include methodological heterogeneity, limited field validation, and underrepresentation of indigenous crops in SSA. The review highlights the urgent need for standardised protocols and expanded molecular capacity. Integrating biochemical markers into breeding programmes may support the development of region-specific resistance screening, and future sustainable nematode management strategies through interdisciplinary collaboration is essential for sustainable nematode control, although further field validation remains necessary. This synthesis offers a foundation to guide future research, crop improvement, and policy decisions under climate stress.

## Background

Food production systems and agricultural sustainability are significantly threatened by root-knot nematodes (*Meloidogyne* spp.), which are among the most economically catastrophic plant-parasitic nematodes in the world (Kantor et al. 2024). Upon invading plant roots, these sedentary endoparasites induce the formation of giant cells, which are hypertrophied cells that impair the plant’s vascular system as well as water and nutrient delivery (Niu et al. 2022). In addition to impairing plant health, the ensuing root galls (often referred to as “root-knots”) increase plant susceptibility to secondary infections (Bernard & Khan 2025). Plants that are infected exhibit withering, chlorosis, stunted growth, and drastically decreased yields (Eisenback and Triantaphyllou 2020; Onkendi et al. 2014; Ramatsitsi et al. 2024). Globally, *Meloidogyne* spp. are contributors to estimated crop losses of 12–14%, with conservative estimates placing yearly crop losses at over US$157 billion (Wong et al. 2021). Losses are often more severe in Sub-Saharan Africa (SSA), where smallholder farming systems predominate and is primarily reliant on susceptible staple crops. Yield losses between 30 and 50% have been reported, especially under monocropping or cultivation on poorly managed soil (Coyne et al. 2018; dos Santos et al. 2019).

Paradoxically, notwithstanding the substantial damage that *Meloidogyne* spp. cause to both the economy and ecological functionality, these nematodes are still often overlooked in research priorities and policy frameworks in SSA (Coyne et al. 2018). In field conditions, infections are frequently misdiagnosed due to symptoms overlap with nutrient deficiencies, drought stress, or other root-related disorders. According to Bogale et al. (2020), since nematodes are microscopic and soilborne, smallholder farmers and extension professionals often lack the specifically tailored sampling and diagnostic expertise and equipment needed for identification. Consequently, *Meloidogyne* spp. exert subtle but cumulative damage, which makes their effects less dramatic (although significantly persistent) than those of apparent and acute pests such as locusts or fungal blights (Khan et al. 2023). Moreover, the development of focussed management programmes in SSA has been hindered by the lack of financial support for nematology in agricultural research, particularly in marginalised countries (Coyne et al. 2018; Karuri et al. 2022). These challenges are further exacerbated by the host range of *Meloidogyne* spp., affecting the majority of SSA staple crops, including maize (*Zea mays*), yam (*Dioscorea* spp.), potato (*Solanum tuberosum*), tomato (*S. lycopersicum*), and cassava (*Manihot esculenta*) (Abdulsalam et al. 2021; Banora and Almaghrabi 2019; Przybylska et al. 2018; Ramatsitsi et al. 2024). Given that the *Meloidogyne* spp. infection can affect any of these crops, they pose a serious biotic threat to household food supply as well as the overall agricultural growth of the region.

Synthetic nematicides have historically been used extensively as conventional nematode management strategy (Tiwari 2024). However, a shift towards sustainable and integrated pest management has proven to be imperative due to concerns about human and ecological health, coupled with growing regulatory constraints and high costs of synthetic treatments (Zhou et al. 2024). Resistant plants, particularly biochemical defence mechanisms, have emerged as a promising alternative (Murungi et al. 2018; Ramatsitsi et al. 2023). When it comes to *Meloidogyne* infestation and infection plants activate a range of biochemical and molecular reactions upon nematode detection. These include the production of reactive oxygen species (ROS), accumulation of phenolic compounds, activation of defence-related enzymes, and the synthesis of secondary metabolites that are nematotoxic (Yang et al. 2018). Moreover, phytohormone signalling is essential in regulating defence gene expression and systemic resistance, particularly through ethylene (ET), salicylic acid (SA), and jasmonic acid (JA) (Mbaluto et al. 2021; Xie et al. 2025). Host responses—whether susceptibility, tolerance, or resistance—reflect the nature and regulation of plant defence mechanisms. In quantitative resistance systems, these outcomes are often influenced by the timing, spatial distribution, and intensity of biochemical and hormonal activation. In contrast, T-gene-mediated resistance involves gene-specific recognition and rapid downstream signalling events.

Although substantial research has been conducted in recent years, the current body of knowledge remains dispersed, particularly regarding the regional variability of host biochemical responses within SSA. Much of the detailed mechanistic understanding derives from studies conducted outside the region, leaving important contextual and methodological gaps in SSA cropping systems. This systematic review aims to synthesise global insights while highlighting SSA-specific evidence to address the questions: (i) What plant biochemical pathways are activated in response to *Meloidogyne* infection, and how do they differ across susceptible, tolerant, and resistant host types? (ii) To what extent are these mechanisms studied in SSA cropping systems, and how are they represented within the broader global evidence base? (iii) Which defence-related compounds and hormonal pathways show the most promise for crop resistance breeding or nematode management?

## Review methodology

### Review framework and protocol

This systematic review was carried out in compliance with the Preferred Reporting Items for Systematic Reviews and Meta-Analyses (PRISMA) guidelines (Page et al. 2021). The PRISMA framework was used to ensure that the review procedure was thorough and rigorous, completely repeatable, and logically organised for the research in interactions between plants and *Meloidogyne* spp. The procedure for this review was created before data extraction and literature search stages. The scope of the review, the criteria for inclusion and exclusion, the search plan, methodology for data extraction, and quality evaluation were all described. Bias in the research selection and result interpretation was reduced by pre-defining these components. Even though the protocol was not registered on PROSPERO (given that it does not include any clinical data), registration was not applicable because PROSPERO is restricted to systematic reviews involving human health-related outcomes. This review focuses on plant–pathogen interactions and agricultural systems. Nonetheless, the protocol was developed a priori, specifying the objectives, eligibility criteria, search strategy, and quality assessment framework to ensure methodological transparency.

With an emphasis on findings from SSA, the review incorporates a global perspective on plant biochemical defence mechanisms against *Meloidogyne* spp. to establish both regional gaps and universal trends. By focussing on experimental research that assesses the biochemical reactions of plants to *Meloidogyne* spp. infestation, this review is grounded in an agroecological and plant-pathological framework. The overall goal was to compile disparate research on metabolic, enzymatic, and molecular signalling pathways that are triggered during nematode assault, especially those linked to nematode growth suppression or resistance. This dual perspective enables the integration of global scientific advancements with region-specific challenges and opportunities in SSA, providing a comprehensive foundation to guide locally adapted management strategies. To logically and purposefully structure the review, the elements in Table 1 were defined and adhered to.

**Table 1:** PICOS framework outlining the criteria used to guide the systematic review of plant biochemical responses to *Meloidogyne* spp. Infestation

Component	Definition	Application in this review
Population	Studied plant species	Crop or non-crop species tested for *Meloidogyne* infection
Exposure	Condition/intervention	Inoculation or natural infestation with *Meloidogyne* spp.
Comparator	Control or reference	Uninfected control plants or know susceptible/resistant genotype
Outcome	Response	Biochemical and molecular changes indicative of a defence response
Study design	Research design	Laboratory, greenhouse, and field-based experimental research

This evaluation excluded narrative reviews, theoretical models, and unsubstantiated assertions in preference for exclusively focussing on research using empirical data. Priority was given to experimental studies that directly measure infected plant biochemical responses following *Meloidogyne* spp. infection. Because of the crucial role these responses play in mediating early plant–nematode interactions and their potential for breeding or biocontrol applications, the focus on biochemical pathways was chosen. Studies that only reported phenotypic symptoms or agronomic impacts without assessing internal plant responses were excluded. A systematic methodology was employed to (i) specify the goals and scope of the review to ensure rigour and coherence, (ii) use an open and reproducible search technique to find all pertinent research, (iii) choose research according to specific inclusion/exclusion criteria, (iv) use a standardised template to extract pertinent data, (v) use a quality grading system to evaluate methodological quality based on predefined methodological criteria, and (vi) where applicable, synthesise and present findings both numerically (e.g. frequency mechanisms) and conceptually (e.g. pathways or host resistance patterns). This rigorous methodology allowed the evaluation to minimise reporting bias, steer clear of subjective selection and preserve uniformity in the assessment of biochemical traits and signalling features across various plant–*Meloidogyne* systems.

### Literature search strategy

Several academic databases and digital repositories were searched for relevant material in a methodical, repeatable manner to ensure thorough coverage and reduce publication bias. The specific objective was to locate experimental research that examined molecular or biochemical defence mechanisms in plants after *Meloidogyne* spp. invasion. In accordance with PRISMA 2020 principles, transparency in reporting, stepwise filtering, and retrieval of pertinent material were highlighted in the search approach. The chosen academic databases were Scopus, Web of Science, PubMed, ScienceDirect, and Google Scholar. These sources were selected to ensure broad disciplinary coverage and applicability to plant pathology, nematology, molecular biology, and agricultural sciences. AGRIS was also searched because of its emphasis on agricultural literature from developing countries, especially SSA. To trace the advancements in molecular methods and phytochemical analyses and capture the most pertinent experimental improvements in plant biochemical responses to *Meloidogyne* spp., no publication date restrictions were applied; however, the included studies spanned the period from 2001 to 2024. Because of limitations on access and linguistic competency, only English-language publications were included.

The search string was created by combining free-text phrases with regulated vocabulary, such as MeSH terms in PubMed. To increase sensitivity of retrieval, wildcards (*) and Boolean operators (AND, OR) were applied strategically. The databases employed the strings presented in Table 2. To improve the review’s precision, the following additional search strategies were adopted: backward citation chaining (reviewing references of included studies), tracking forward citations using search databases, and open archives such as FAO for agricultural reports, theses, and institutional research to find possibly pertinent non-journal contributions. All search results were exported into EndNote reference manager. Before being deleted, duplicate records were automatically identified through EndNote and manually verified. Following this, each article was passed through two screening process: (i) title and abstract screening to determine preliminary relevance and (ii) full-text screening in accordance with the predefined inclusion/exclusion criteria outlined in Table 2.

**Table 2:** Summary of search strings used for the systematic review

Database	Search string	Fields	Filters applied
Scopus	[TITLE-ABS-KEY (“*Meloidogyne*” OR “root-knot nematode”)] AND [TITLE-ABS-KEY (“plant defence” OR “plant defense” OR “resistance” OR “tolerance” OR “susceptible” OR “biochemical response” OR “secondary metabolites” OR “enzyme activity” OR “phenolics” OR “reactive oxygen species” OR “salicylic acid” OR “jasmonic acid” OR “flavonoids”)] AND [TITLE-ABS-KEY (“experimental study” OR “greenhouse” OR “laboratory” OR “field study”)]	Title, abstract, keywords	Research articles, English
Web of Science	[TS (“*Meloidogyne*” OR “root-knot nematode”)] AND [TS (“plant defence” OR “plant defense” OR “resistance” OR “tolerance” OR “susceptible” OR “biochemical response” OR “secondary metabolites” OR “enzyme activity” OR “phenolics” OR “reactive oxygen species” OR “salicylic acid” OR “jasmonic acid” OR “flavonoids”)] AND [TS (“experimental study” OR “greenhouse” OR “laboratory” OR “field study”)]	Topic (TS)	Research articles, English
PubMed	[Title/Abstract (“*Meloidogyne*” OR “root-knot nematode”)] AND [Title/Abstract (“plant defence” OR “plant defense” OR “resistance” OR “tolerance” OR “susceptible” OR “biochemical response” OR “secondary metabolites” OR “enzyme activity” OR “phenolics” OR “reactive oxygen species” OR “salicylic acid” OR “jasmonic acid” OR “flavonoids”)] AND [Title/Abstract (“experimental study” OR “greenhouse” OR “laboratory” OR “field study”)]	Title/Abstract	Journal articles, English
Science Direct	(“*Meloidogyne*” OR “root-knot nematode”) AND (“plant defence” OR “plant defense” OR “resistance” OR “tolerance” OR “susceptible” OR “biochemical response” OR “secondary metabolites” OR “enzyme activity” OR “phenolics” OR “reactive oxygen species” OR “salicylic acid” OR “jasmonic acid” OR “flavonoids”)] AND (“experimental study” OR “greenhouse” OR “laboratory” OR “field study”)	Title, abstract, keywords	Peer-reviewed, English
Google scholar	“*Meloidogyne*” AND “plant biochemical response” AND “enzyme activity” OR “secondary metabolites” OR “plant defence” OR “plant defense”	Full text	Journal articles, English

### Inclusion and exclusion criteria

During the screening procedure, a predetermined set of inclusion and exclusion criteria were applied to all retrieved papers to guarantee a targeted and thorough synthesis. These standards were created to find empirical studies that, through experimental validation, examined the molecular defence mechanisms of plants after *Meloidogyne* spp., including biochemical, physiological, and/or molecular mechanisms following infection. Eligible studies were required to report quantifiable outcome measures such as gall index, nematode RF, egg mass counts, infection severity, or associated defence-related biochemical or gene expression parameters. Studies were excluded if they lacked experimental validation, did not assess host defence responses, focussed solely on agronomic performance without mechanistic analysis, or did not involve *Meloidogyne* spp. infection assays. Reviews, meta-analyses, and purely descriptive studies without experimental infection data were excluded. To reduce selection bias, the criteria were used methodically by one author, while the other verified, during the screening of titles, abstracts and full texts. Studies were included if they met all the conditions stipulated in Table 3.

**Table 3:** The criteria for selection of articles for host response to *Meloidogyne* spp.

Criteria	Inclusion	Exclusion
Target organism	*Meloidogyne* spp. (e.g. *M. incognita*, *M. javanica*, and *M. enterolobii*)	Other plant-parasitic nematodes (e.g. *Pratylenchus* spp., *Heterodera* spp., and *Globodera* spp.)
Focus	Plant biochemical defence mechanisms (e.g. secondary metabolites, enzymes, and hormones)	Studies without biochemical data (e.g. only morphological responses or yield-based assessment)
Study design	Experimental studies (e.g. laboratory, greenhouse, and field)	Reviews, modelling studies, descriptive studies, opinion papers
Data type	Reports measurable biochemical or metabolic outcomes	No quantifiable biochemical measurements
Stress condition	*Meloidogyne*-induced responses isolated or clearly interpretable	Combined stressor studies without isolating *Meloidogyne*-specific effects
Publication type	Peer-reviewed articles in English without date restriction	Non-English articles, conference abstracts

### Study selection process

The PRISMA 2020 standards (Page et al. 2021) were adhered to during the study selection process to ensure scientific rigour, transparency, and reproducibility. To reduce selection bias and ensure consistency, a structured multi-stage procedure with three stages was carried out. Each stage was carried out systematically by the primary author and validated by the secondary author to ensure inter-reviewer reliability.

*Stage 1: Title verification*—Duplicate records were found and eliminated once all records were obtained from the databases and imported into EndNote. Following this, titles were screened to exclude studies that were outside the scope of the review, such as those that focussed on non-*Meloidogyne* spp. or had nothing to do with plant–nematode interactions. At this point, studies that did not include any kind of physiological or biochemical response in plants were also excluded. To prevent premature exclusion of potentially pertinent research, the screening was purposefully wide. To reduce false negatives, borderline titles were retained and advanced to the abstract screening stage.

*Stage 2: Abstract screening*—The established inclusion and exclusion criteria were used to screen the abstracts of the remaining papers. Articles with an abstract indicating an emphasis on experimental investigation of plant responses to *Meloidogyne* spp. were kept. The studies that mentioned biochemical indicators (e.g. enzymatic assays) or experimental modification of defence mechanisms received special attention. On the other hand, studies lacking specificity regarding the mechanistic nature of plant responses or those presenting purely observational findings without biochemical metrics were excluded. To improve accuracy, abstracts were independently reviewed by both the authors.

**Fig. 1:**
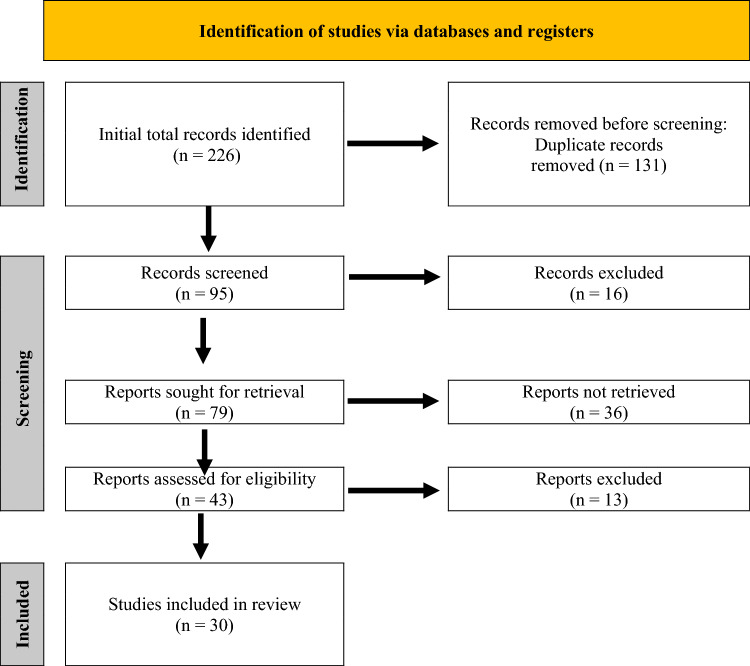
PRISMA flow diagram for eligible articles for inclusion in the systematic review

*Stage 3: Full-text screening*—Eligible abstracts were retrieved and subjected to comprehensive full-text assessment. Only studies that reported measurable biochemical results in response to *Meloidogyne* invasion and included adequate methodological detail were retained. Articles were excluded if the nematode effect could not be isolated, such as in cases of mixed biotic–abiotic stress unless the *Meloidogyne*-specific results were clearly identifiable. To standardised full-text examinations, a screening checklist based on PICOS (population, intervention, comparator, outcomes, study design) framework was employed. A detailed record of reasons for excluding full-text articles was kept. All screening steps were documented and managed using Microsoft Excel for traceability. A PRISMA flow diagram was created to document the full trajectory of the study identification, screening, eligibility assessment, and final inclusion (Fig. 1).

### Data extraction and synthesis

Relevant information was carefully gathered from included studies using a standardised data extraction approach. In addition to recording experimental results, the objective was to record contextual information required for theme synthesis, biochemical reaction comparisons, and methodological qualified evaluation. A customised data extraction form was created in Microsoft Excel. After the programme was refined, data were extracted from every full-text article that was included. Structured synthesis across species, chemical, and defence pathways was made possible by this thorough extraction, and it served as the foundation for the comparative analysis in the findings and discussion sections. Host responses were categorised as resistant, tolerant, or susceptible based on outcome metrics reported in each study, including gall index, nematode reproduction factor (RF), egg mass counts, infection severity, and plant performance indicators. Where authors explicitly defined host categories, their classification was retained. In studies where categorical labels were not provided, classification was inferred from relative differences in nematode reproduction and plant growth compared with controls. Due to heterogeneity in experimental systems and reporting standards, no universal numerical thresholds were imposed; instead, classifications were based on within-study comparisons. In classical plant pathology, resistance is often associated with R-gene-mediated recognition mechanisms, whereas tolerance is typically quantitative and polygenic, and susceptibility reflects the absence or ineffectiveness of resistance mechanisms. However, not all included studies genetically characterised the underlying basis of resistance. Therefore, the terminology used in this review reflects reported phenotypic and experimental outcomes rather than confirmed genetic architecture. Analytical approaches were categorised as spectrophotometric enzyme assays, chromatographic metabolite analyses, or molecular/omics-based techniques to distinguish methodological depth and resolution.

## Results

### Overview of included studies

This review synthesises findings from 30 experimental studies conducted across 17 countries, focussing on plant biochemical defence responses to *Meloidogyne* spp. infections. Of these, five studies originated from SSA, with Kenya contributing four studies (Kihika et al. 2017; Murungi et al. 2018; Kihika-Opanda et al. 2022; Mwamba et al. 2021) and Nigeria contributing one study (Egedigwe et al. 2024), reflecting a growing regional effort. The dominance of Kenyan studies aligns with the country’s status as a recognised centre for nematology research, supported by long-standing programmes at institutions such as the International Centre of Insect Physiology and Ecology (ICIPE) and Kenya Agricultural and Livestock Research Organization (KALRO) (Nature 2016). In contrast, the remaining 24 studies were conducted in other regions including countries such as Belgium (Nahar et al. 2011, 2013; Ji et al. 2015; Kyndt et al. 2016; Singh et al. 2019; Chavan et al. 2022), India (Rani et al. 2008; Abbasi & Hisamuddin 2014; Singh et al. 2020; Danish et al. 2021), China (Yang et al. 2018; Sikandar et al. 2024), the USA (Goellner et al. 2001; Branch et al. 2004), Brazil (Castaneda et al. 2017), and Iran (Moslemi et al. 2016). These studies often focussed on genetically tractable model crops such as Arabidopsis (*Arabidopsis thaliana*) and tomato, and typically employed advanced tools including transcriptomics, hormone profiling, and mutant lines, offering a molecularly rich but geographically narrow view of plant–nematode biochemical interactions.

Of the 30 included studies, 1 was conducted under field conditions, while the remainder were performed under laboratory, greenhouse, or controlled growth chamber settings. Studies across African regions more frequently employed in vitro assays and focussed on underutilised or indigenous crops such as black nightshade (*S*. *nigrum* L.), okra (*Abelmoschus esculentus* L. Moench), and African pepper (*Piper guineense* Schumach. & Thonn.). These studies prioritised natural product-mediated resistance, including volatile organic compounds, root exudates, and antioxidant enzyme responses, reflecting a strong biocontrol and low-cost innovation focus. Despite South Africa’s documented prominence in nematology research output (Bowman and Chisoro 2024; Habiyaremye 2024), no experimental studies from the country met the biochemical and mechanistic inclusion criteria of this review. This does not suggest absence of nematology research, but rather highlights a specific gap in experimentally validated plant biochemical defence studies against *Meloidogyne* spp. This suggests a potential disconnect between national nematology programmes and biochemical resistance screening, and highlights a valuable opportunity for future, targeted investigations. Overall, the dataset reflects both the diversity of methodological approaches used globally and the regional disparities in research funding, capacity, and crop focus, all of which shape the current understanding of plant biochemical defences against *Meloidogyne* spp.

### Biochemical defence mechanisms identified

The diverse range of biochemical defensive responses that plants displayed following infection by *Meloidogyne* spp. in the 30 experimental trials that were analysed illustrates complex molecular reprogramming. The most reported pathways included phenolic compound synthesis (e.g. Moslemi et al. 2016; Abed et al. 2023), ROS accumulation (e.g. Ji et al. 2015; Yang et al. 2018), phytohormonal modulation, particularly involving ET, SA, and JA (e.g. Mbaluto et al. 2021; Sikder et al. 2021) and increased expression and/or defence-related enzymes such as peroxidase (POD), polyphenol oxidase (PPO), phenylalanine ammonia-lyase (PAL), and superoxide dismutase (SOD) (e.g. Yang et al. 2018; Singh et al. 2019). The production of ROS, namely hydrogen peroxide (H_2_O₂) and superoxide radicals (O₂⁻), was a nearly common response during the early stages of nematode invasion. Such compounds function as direct antibacterial agents as well as secondary signalling messengers that activate downstream defences. Enzymatic antioxidants like SOD, catalase (CAT), and ascorbate peroxidase (APX) were routinely evaluated to assess oxidative stress equilibrium (Afifi et al. 2014; Egedigwe et al. 2024). Most studies found that sensitive cultivars had uncontrolled ROS production and root damage (Goellner et al. 2001; Abbasi & Hisamuddin 2014), while resistant lines displayed a regulated oxidative burst along with antioxidant responses (Kihika-Opanda et al. 2022; Abed et al. 2023).

Roots under nematode exposure had greater levels of phenolic compounds, including flavonoids, lignin precursors, and total phenols. These compounds serve as substrates for the oxidative crosslinking and lignification of proteins, strengthening cell walls and preventing juveniles from penetrating root tissues. PAL activity, a key enzyme in the phenylpropanoid pathway, was a consistent biomarker of this reaction, especially in tomato cv. ‘Money Maker’ from SSA (Kihika-Opanda et al. 2022) and banana (*Musa acuminata* cv. ‘Pisang Mas’) from Brazil (Castaneda et al. 2017). Hormonal response reports ranged widely. Studies such as Branch et al. (2004), Santos et al. (2013), and Yang et al. (2018) reported on SA signalling commonly associated to SAR, and was more prominent in resistant cultivars, but JA and ET were linked to ISR pathways, particularly in monocots (Nahar et al. 2011). Across the included studies, several methodological tendencies became apparent. Research conducted in SSA has frequently focussed on quantitative assessments of POD and PAL activity, total phenolics, and, in some cases, ROS dynamics, while chromatographic metabolite profiling and molecular or omics-based approaches have been increasingly adopted in other regions. This pattern likely reflects differences in methodological focus and available analytical platforms across research settings (Kihika et al. 2017; Mwamba et al. 2021; Kihika-Opanda et al. 2022). However, studies carried out throughout the world, especially in China, Brazil, and India, increasingly frequently used metabolomics, transcriptome analysis, and hormonal profiling (Castaneda et al. 2017; Yang et al. 2018; Singh et al. 2020), which made it possible to understand regulatory crosstalk and pathway linkages in greater depth.

### Analytical techniques and experimental approaches

Several analytical techniques and experimental strategies were employed in the reviewed studies to investigate the biochemical responses of plants to *Meloidogyne* spp. infections. Analytical approaches ranged from colorimetric enzyme assays to molecular and chromatographic techniques. The variability in technique options was influenced by the specific study topic, crop kind, and regional capabilities. For detecting biochemical reactions such as PAL, POD, PPO, SOD, and CAT, spectrophotometric enzymatic assays were the most used analytical technique. These assays were widely employed across the included studies due to their established protocols, reproducibility, and effectiveness in quantifying enzymatic defence responses. Remarkably, the majority of SSA research mainly depended on these colorimetric techniques, frequently using standard substrates such as guaiacol (for POD), catechol (for PPO), nitroblue tetrazolium (for SOD), and employed chromatographic techniques such as gas chromatography–mass spectrometry (GC–MS) (Murungi et al. 2018). However, research from throughout the world, particularly that from Brazil, China, India, and some regions of Europe, demonstrated a higher level of analytical complexity. To identify and measure specific phytohormones, secondary metabolites, or phenolic compounds (such as SA, JA, or ABA), these investigations commonly employed spectrophotometric enzyme assays (Abed et al. 2023) and high-performance liquid chromatography (HPLC) (Mbaluto et al. 2021). These methods allowed for more accurate and molecularly detailed correlations between certain metabolic pathways and biological processes.

### Outcomes: host susceptibility, tolerance and resistance

The 30 experimental studies that included host responses to *Meloidogyne* spp. infections were categorised into resistant (47%), tolerant (9%), and susceptible (44%) outcomes. A total of 15 plant entries demonstrated resistant responses, with 7 originating from SSA and 8 from non-SSA regions. Resistance in SSA studies was largely driven by the production of nematode-repelling volatiles and root exudates, such as α-pinene, limonene, and β-caryophyllene in blackjack (*Bidens pilosa* L.), spinach (*Spinacia oleracea* L.), and various Asteraceae species (Murungi et al. 2018; Mwamba et al. 2021; Kihika-Opanda et al. 2022). These compounds significantly reduced nematode attraction and hatchability, indicating potent biochemical deterrence mechanisms. In contrast, resistance in non-SSA plants was predominantly associated with systemic acquired resistance (SAR), induced systemic resistance (ISR), and hormone-regulated signalling involving SA, JA, and ET. For instance, rice (*Oryza sativa* cv. ‘Nipponbare’) and tomato cv. ‘Motelle’ exhibited enhanced SA and JA pathway activation, phenylpropanoid build-up, and callose deposition, effectively suppressing galling and nematode development (Branch et al. 2004; Chavan et al. 2022; Nahar et al. 2011; Singh et al. 2020; Qi et al. 2026).

Tolerant responses were comparatively rare, occurring in only three plants, one from SSA (Egedigwe et al. 2024) and two from Asia (Moslemi et al. 2016; Abed et al. 2023). Tolerance was typically linked to oxidative burst and osmotic adjustment, as seen in okra cv. ‘Meya’ and tomato cv. ‘Early Urbana’, where elevated levels of proline, H₂O₂, and antioxidant enzymes like peroxidase and superoxide dismutase were observed (Moslemi et al. 2016; Egedigwe et al. 2024). While these responses did not fully inhibit nematode reproduction, they mitigated physiological damage and reduced gall severity. A similar pattern was seen in cucumber (*Cucumis sativus* cv. ‘Babylon’), which showed partial biochemical activation without complete resistance (Abed et al. 2023). Tolerance should be distinguished from partial resistance in that nematode reproduction may occur at levels comparable to susceptible hosts; however, the plant maintains growth performance or exhibits compensatory physiological adjustments that mitigate yield loss.

Fifteen studies classified plants as susceptible, with comparable distribution between SSA (7 plants) and non-SSA (8 plants). In SSA, susceptibility was often attributed to attractant volatile emissions, such as methyl salicylate in *Capsicum annuum* cultivars, and compromised JA signalling, particularly in tomato cv. ‘Money Maker’ (Kihika et al. 2017; Murungi et al. 2018; Mbaluto et al. 2021). Non-SSA studies reported similar trends, where Arabidopsis and mung bean (*Vigna radiata*) showed weak or suppressed defence responses despite some antioxidant activation (Goellner et al. 2001; Abbasi & Hisamuddin 2014). Notably, rice under brassinosteroid treatment exhibited hormone crosstalk that antagonised JA-mediated defences, rendering the plants more vulnerable to *M. graminicola* (Nahar et al. 2013). Other studies also reported upregulation of nematode-susceptibility-associated genes, as in banana and cucumber cv. ‘Nadeen’ (Castaneda et al. 2017; Abed et al. 2023).

**Fig. 2:**
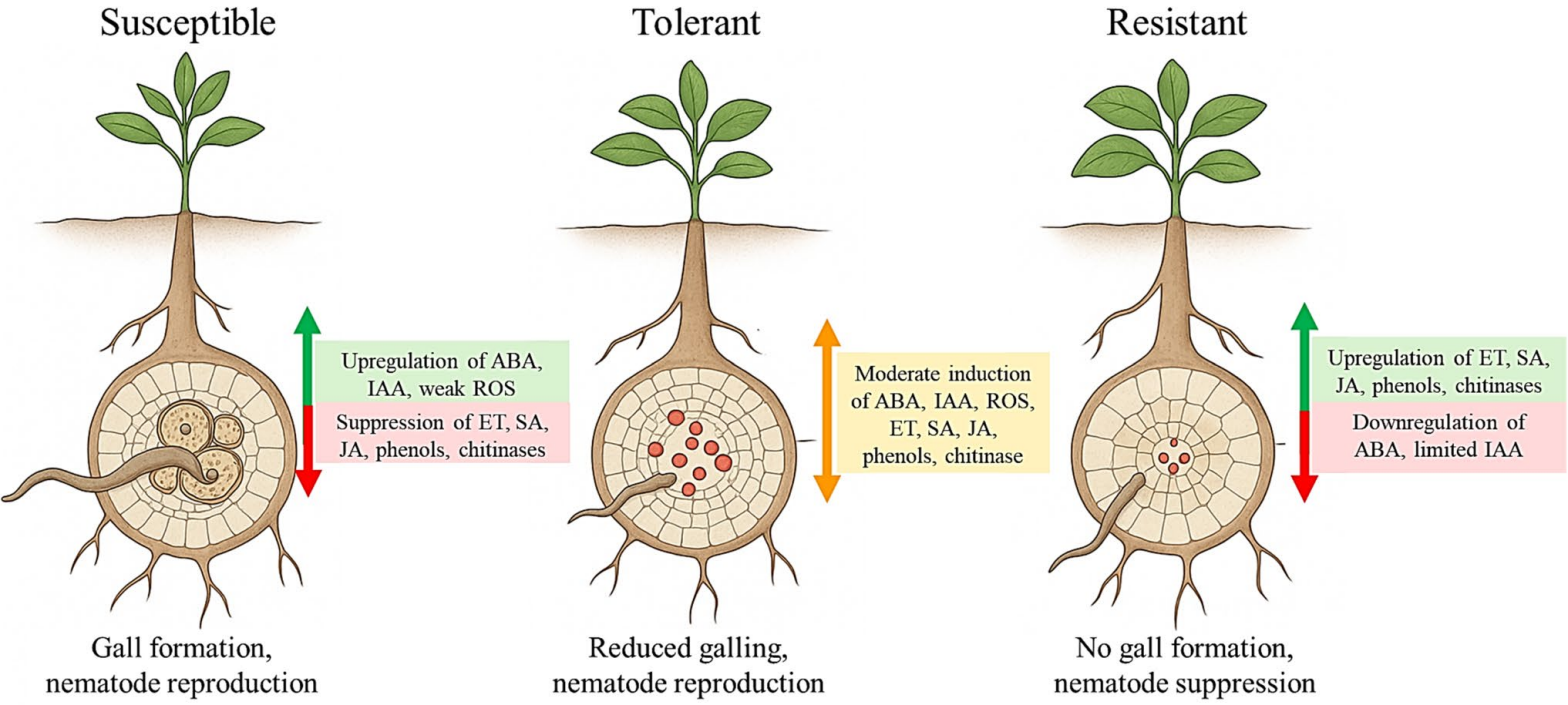
Schematic representation of biochemical and hormonal responses in susceptible, tolerant, and resistant plant hosts following *Meloidogyne* spp. infection. The schematic includes reactive oxygen species (ROS) dynamics, antioxidant enzyme activity (e.g. SOD, CAT, and POD), phenylpropanoid pathway activation (PAL and phenolic accumulation), cell wall reinforcement, and SA/JA/auxin-associated signalling pathways. In susceptible plants, nematodes suppress or evade early defences, redirect auxin to promote gall formation, and downregulate SA/JA signalling, leading to high reproduction. Tolerant plants exhibit moderate antioxidant and hormonal responses that contain damage without fully restricting infection. Resistant plants rapidly activate ROS bursts, reinforce cell walls, and upregulate SA, JA, and PR proteins to block nematode development. Host categories are visually distinguished by [colour coding (green for upregulation, red for suppression and orange for moderate induction of hormones/enzymes)], with arrows indicating relative activation or suppression of pathways. These profiles highlight biochemical gradation in host response outcomes and reflect findings across global and Sub-Saharan African cropping systems.

Overall, SSA studies revealed a distinct emphasis on biochemical repellents and allelopathic traits, while non-SSA research more frequently explored intracellular signalling, hormone crosstalk, and transcriptional regulation. Tolerance remains underexplored and mechanistically intermediate, underscoring the need for more comparative studies across genotypes and regions. Figure 2 is an illustration of host responses to *Meloidogyne* spp. Infection (Table 4).

**Table 4:** Summary of experimental studies evaluating plant biochemical defences against *Meloidogyne* spp.

Plant	Outcome	Country	Target nematode	Experimental setup	Plant defence mechanisms	Biochemical markers	Analytical techniques	Source
*C. annuum* cvs. ‘California Wonder’, ‘Yolo Wonder’, ‘Long red Cayenne’	Susceptible (increased attraction + galling)	Kenya	*M. incognita*	In vitro dual-choice chemotaxis bioassay	Host location mediated by volatile cues; attractant chemotaxis	Methyl salicylate (MeSA)	GC–MS + chemotaxis assay	(Kihika et al. 2017)
*S. lycopersicum* cv. ‘Money Maker’	Susceptible	Kenya	*M. incognita*	In vitro	Attraction	2-Isopropyl-3-methoxypyrazine, 2-(methoxy)−3-(1-methylpropyl) pyrazine, tridecane, α- and β-cedrene, δ−3-carene, sabinene, and methyl salicylate	SPME, GC–MS	(Murungi et al. 2018)
*A. thaliana*	Susceptible (reduced by SA and JA)	Denmark	*M. incognita*	In vitro	Hormone-specific responses: SA and JA restricted infection; ABA and auxin	SA, JA, ET, auxin, ABA pathways	Nematode attraction and infection assays	(Sikder et al. 2021)
*S*. *lycopersicum* cv. ‘Money Maker’	Susceptible (suppressed JA-related leaf defences)	Netherlands	*M. incognita*	Greenhouse	Suppression of JA-mediated anti-herbivore defences due to nematode-induced SA signalling	SA, JA, OPDA, JA-Ile, ABA, α-tomatine	LC–MS/MS for phytohormone profiling; qPCR for gene expression; HPLC for α-tomatine	(Mbaluto et al. 2021)
*Cucurbita pepo* L	Susceptible	Egypt	*M. incognita*	Pot + field trials	Weak activation of antioxidant defence	Ascorbic acid, CAT, phenols, POD, polyphenoloxidase, APX	Spectrophotometry	(Afifi et al. 2014)
*O. sativa* cv. 'Nipponbare'	Susceptible (BR-JA antagonism-modulated susceptibility)	Belgium	*M. graminicola*	Growth chamber	Brassinosteroids suppressed JA-mediated defence	Decreased JA-response genes	qRT-PCR	(Nahar et al. 2013)
*M. acuminata* cv. 'Pisang Mas'	Susceptible	Brazil	*M. incognita*	Greenhouse	Enhanced expression of nematode-susceptibility-associated genes	Downregulated PR proteins, PAL, lipoxygenase, with upregulated cell wall-modifying genes	RNA-seq, qRT-PCR	(Castaneda et al. 2017)
*S. lycopersicum* cv. ‘Zhongza 09’	Susceptible (triggered stress response enzymes, increased nematode density)	China	*M. enterolobii*	Pot trial with graded nematode levels	Induced oxidative stress response	POD, CAT, malondialdehyde	Enzyme assay	(Sikandar et al. 2024)
*Trachyspermum ammi*	Susceptible (physiological damage and structural alterations)	India	*M. incognita*	Pot trial in glasshouse	Antioxidant defence response, cellular stress adaptation	Increased POD, SOD, CAT	Enzyme assay	(Danish et al. 2021)
*A. thaliana* Col-0	Susceptible (manipulated by nematode to favour infection via auxin redistribution)	Belgium	*M. incognita*	In vitro	Infection caused redirection of auxin flow towards feeding sites	IAA, PIN protein localisation, DR5:GUS expression	Histochemical GUS staining, confocal microscopy, qRT-PC	(Kyndt et al. 2016)
*A. thaliana*	Susceptible (Induction of host endo-glucanase expression to aid in giant cell formation)	USA	*M. incognita*	In planta gene expression study	Modulation of host cell wall metabolism favouring establishment of feeding site	Endo-β−1, 4-glucanase	In situ hybridisation, GUS staining, transcript profiling	(Goellner et al. 2001)
*Pennisetum glaucum* (resistant and susceptible hybrids)	Susceptible hybrid showed weaker biochemical response; resistant hybrid showed enhanced defence enzyme activity and lower nematode reproduction	India	*M. graminicola*	Pot trial	Differential defence activation in resistant hybrids vs. susceptible hybrids; accumulation of defence enzymes and oxidative stress modulation	POD, PPO, CAT, phenols, protein	Enzymatic assays, protein estimation	(Singh et al. 2020)
*V. radiata*	Susceptible (upregulation of antioxidant enzymes + progressive decline in growth)	India	*M. incognita*	Pot trial	Induced oxidative and enzymatic defence under nematode response	POD, PPO, phenols	Spectrophotometry for biochemical estimation	(Abbasi & Hisamuddin 2014)
*S. lycopersicum* cvs. ‘Motelle’ and ‘Money Maker’	‘Money Maker’ and SA-deficient plants were highly susceptible; resistant (‘Motelle’ plants showed strong resistance dependent on SA)	USA	*M. incognita*	Growth chamber	SA signalling	SA, PR-1 gene expression	SA quantification (spectrophotometry), RT-PCR for gene expression, nematode reproduction assays	(Branch et al. 2004)
*S. lycopersicum* (formerly *Lycopersicon esculentum*) **cvs.** ‘Pusa Ruby’, ‘Pusa Early Dwarf’, ‘PKM-1’	Cultivar-dependent response: cvs. ‘Pusa Ruby’, ‘Pusa Early Dwarf’ were susceptible; cv. PKM-1 was resistant	India	*M. incognita*	Greenhouse trial	ISR, enhanced lignification and enzymatic defence	Total phenols, POD, PPO, PAL	Spectrophotometry for enzymes assays	(Rani et al. 2008)
*C. sativus* cvs. ‘Negeen’, ‘Babylon’, ‘Nadeen’	‘Nadeen’—susceptible; ‘Babylon’—tolerant ‘Negeen’—resistant	Iraq	*Meloidogyne* spp.	Greenhouse	Activation of antioxidative and stress-related enzymes	Total phenols, POD, PPO, ascorbic acid, malondialdehyde	Spectrophotometric enzyme assays and biochemical quantification	(Abed et al. 2023)
*A. esculentus* cv. ‘Meya’	Tolerant (enhanced enzymatic activity)	Nigeria	*M. incognita*	Greenhouse	Oxidative burst, osmotic adjustment, antioxidant defence	H_2_O_2_, proline, phenolics, POD, superoxidase dismutase	Spectrophotometry-based enzymatic assay	(Egedigwe et al. 2024)
*S. lycopersicum* cv. ‘Early Urbana’	Tolerant (reduced galling and nematode reproduction)	Iran	*M. javanica*	Greenhouse	SAR	SA; enhanced phenolic content; lignin formation	Root gall index, egg mass counts, phenolic quantification, histochemical staining	(Moslemi et al. 2016)
*S. nigrum*	Resistant (suppressed infestation)	Kenya	*M. incognita*	Intercrop + pot assay (in vitro + greenhouse)	Root exudate bioactive suppression	Vitamins, aromatic acid, flavonoid	Chemical fractionation + in vitro hatching assay, GC–MS	(Kihika-Opanda et al. 2022)
*B. pilosa*	Resistant (significant nematode repellent + egg hatch inhibition)	Kenya	*M. incognita*	In vitro	VOCs + root exudates	α-Pinene, β-caryophyllene, β-selinene, germacrene D	GC–MS	(Kihika-Opanda et al. 2022)
*S. lycopersicum* cv. ‘Money Maker’	Reduced galling + nematode population density	Kenya	*M. incognita*	Intercrop + pot assay	Induced resistance through biochemical priming from *B. pilosa*	Phenols, antioxidant enzymes	Spectrophotometry, enzymatic assay	(Kihika-Opanda et al. 2022)
*C. annuum* cv. AVDRC PP0237	Resistant (reduced attraction + galling)	Kenya	*M. incognita*	In vitro dual-choice chemotaxis bioassay	Host location mediated by volatile cues; repellent chemotaxis	α-Pinene, limonene, 2-methoxy-3-(1-methylpropyl)-pyrazine, methyl salicylate tridecane, thymol	GC–MS + chemotaxis assay	(Kihika et al. 2017)
Asteraceae (*Aspilia pluriseta, A*. *conyzoides, Emilia sonchifolia, Vernonia lasiopus*)	Resistant (significant avoidance behaviour by *M. incognita*)	Kenya	*M. incognita*	In vitro	Repellent by VOCs	Limonene, α-pinene, β-caryophyllene	GC–MS + headspace volatile collection	(Mwamba et al. 2021)
*S. oleracea* cv. ‘Fordhood Giant’	Resistant	Kenya	*M. incognita*	In vitro	Repellent	2-Isopropyl-3-methoxypyrazine, 2-(methoxy)−3-(1-methylpropyl) pyrazine, tridecane, and α- and β-cedrene	SPME, GC–MS	(Murungi et al. 2018)
*O. sativa* cv. ‘Nipponbare’	Resistant (reduced nematode invasion and gall formation)	Belgium	*M*. *graminicola*	Hydroponic	JA, SIR	JA, OsAOS2, OsJAZ9, PR genes	qRT-PCR for gene expression; GUS staining	(Nahar et al. 2011)
*S. tuberosum* cv. ‘Désirée’ *S. lycopersicum* cv. ‘Money Maker’	Resistant (reduction in egg masses and galling)	Portugal	*M*. *chitwoodi*	In vitro + greenhouse	ISR, SAR	SA, JA pathways; BABA-induced priming; chitosan-triggered ISR	Nematode egg mass counts; gall index	(Santos et al. 2013)
*O*. *sativa* cv. ‘Nipponbare’	Resistant (DHA-induced)	Belgium	*M. graminicola*	Laboratory + greenhouse assays	DHA primed JA and ET pathways	DHA, JA, ET, PR1a, OsACS1, OsAOS2	qRT-PCR for gene expression; root staining + microscopic observation	(Chavan et al. 2022)
*Citrullus lanatus*	Resistant (red light significantly reduced galling and nematode reproduction)	China	*M. incognita*	Greenhouse	Red light exposure enhanced systemic resistance via crosstalk between SA, JA, and redox signalling	SA, JA, H₂O₂, ascorbate, glutathione, SOD, CAT, POD, PR1	HPLC for hormones; spectrophotometric assays for ROS and enzymes; microscopic observations	(Yang et al. 2018)
*O*. *sativa*	Resistant (reduced gall formation and nematode development)	Belgium	*M. graminicola*	Greenhouse	BABA triggered enhanced basal defence; no SA/JA/ET priming observed; resistance linked to callose deposition and ROS	Callose, H₂O₂, phenolics	Histochemical staining (aniline blue for callose, DAB for H₂O₂)	(Ji et al. 2015)
*O*. *sativa*	Resistant (suppressed nematode infection and galling)	Belgium	*M. graminicola*	Greenhouse	Systemic acquired resistance; induced phenylpropanoid pathway stimulate secondary metabolite build-up	Phenolics, lignin, PAL, POD	Enzymatic assays (PAL, POD), histochemical staining for lignin	(Singh et al. 2019)

*GC/MS* chromatography/mass spectrometry, *SPME* solid-phase microextraction, *DHA* dehydroascorbate, *ET* ethylene, *POD* peroxidase, *PPO* polyphenol oxidase, *PAL* phenylalanine ammonia-lyase, *SOD* superoxide dismutase, *CAT* catalase, *APX* ascorbate peroxidase, *PR* pathogenesis-related

## Discussion

### Synthesis of key findings

This systematic investigation provides a comprehensive synthesis of plant biochemical defence mechanisms against *Meloidogyne* spp. from both SSA and global perspectives. Our analysis of 30 experimental studies yielded several significant findings. *Meloidogyne incognita* remains the most often researched *Meloidogyne* spp. worldwide, especially in SSA, which is indicative of its extensive range and serious risk to major crops. The uneven geographic distribution of research, with SSA offering fewer studies, highlights regional disparities in research capacity and resource availability. Nonetheless, the expanding quantity of SSA research suggests that local agricultural issues are receiving more scientific attention. Commonly observed biochemical responses to *Meloidogyne* invasion include the accumulation of ROS, the build-up of more phenolic compounds, and the activation of defence-related enzymes, including POD and PAL. These responses are facilitated by intricate phytohormonal signalling networks, namely the ET, SA, and JA pathways.

While global studies including those by Kyndt et al. (2016) and Chavan et al. (2022) typically use chromatographic metabolite profiling and molecular or omics-based analyses techniques to fully understand these mechanisms, most SSA research such as Kihika et al. (2017) and Egedigwe et al. (2024) uses spectrophotometric enzyme assays, which reflect infrastructure limitations but also provide important context-specific insights into native crop responses. Furthermore, this review demonstrates that resistant host plants often exhibit early and robust defence enzyme and secondary metabolite activation, which is linked to reduced gall formation and nematode population growth. On the other hand, *Meloidogyne* invasion of susceptible crops results from inadequate or delayed biochemical responses. It was evident that tolerance, a less often reported response, involves a relatively small amount of metabolic activity that reduces damage without completely inhibiting nematode survival. These findings collectively demonstrate that plants respond to *Meloidogyne* spp. with a unique biochemical “warfare” that varies slightly by crop and area. Apart from validating established defensive paradigms, the findings gathered underscore the need for greater methodological sophistication and capacity building in SSA to fully comprehend and leverage indigenous crop resistance mechanisms.

### Biochemical defence pathways in diverse cropping systems

Complex biochemical networks are involved in plant responses to *Meloidogyne* infection, which are initiated by nematode detection. These include pathogenesis-related (PR) protein expression, cell wall remodelling, oxidative stress control, secondary metabolite generation, and hormonal interaction, according to research conducted worldwide. The majority of this knowledge is derived from model crops such as Arabidopsis, rice, and tomato, but new research from SSA offers unique perspectives on understudied crops, resistance mediated by natural products, and low-input techniques (Mwamba et al. 2021; Egedigwe et al. 2024).

**Fig. 3:**
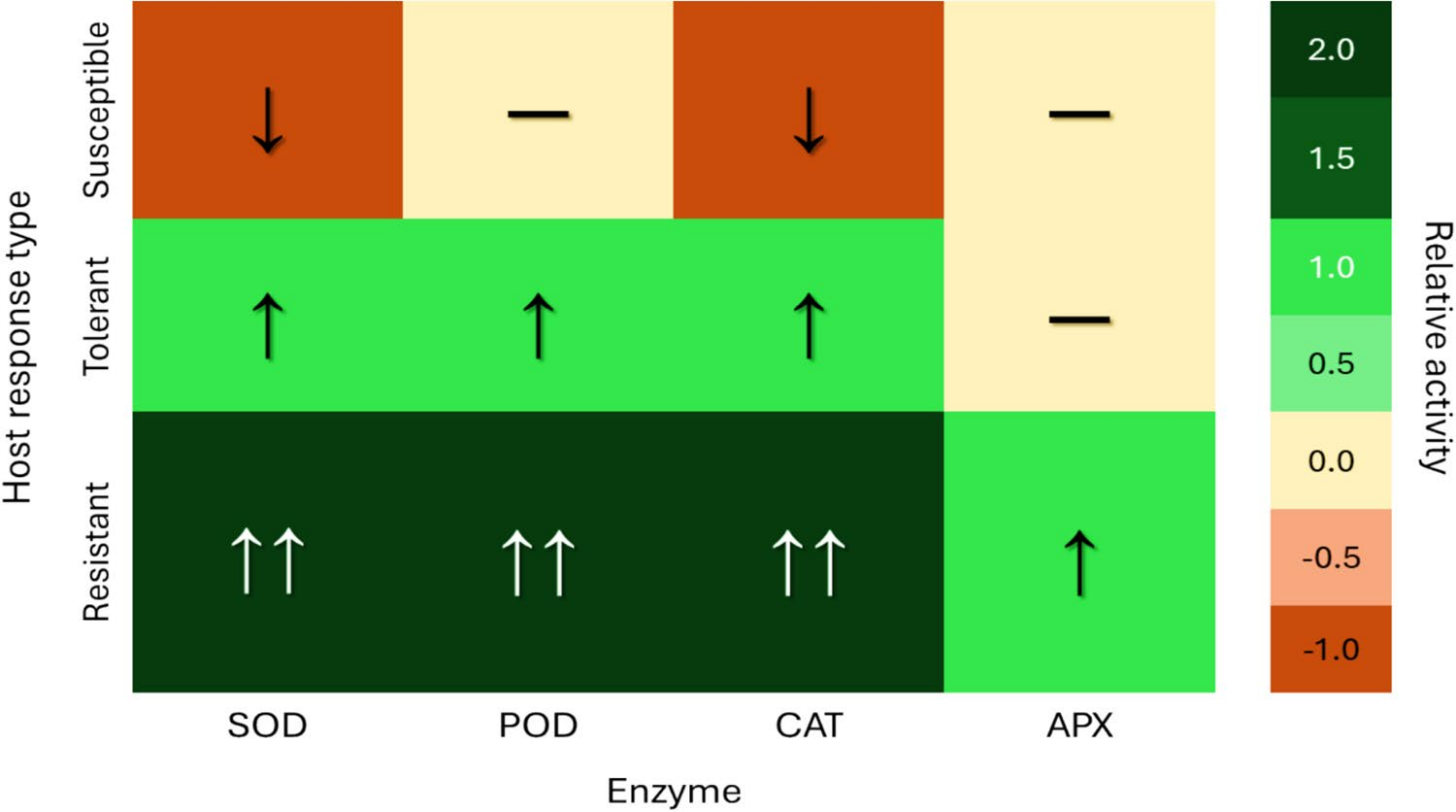
Qualitative heatmap of antioxidant enzyme activity in susceptible, tolerant, and resistant plant hosts after *Meloidogyne* spp. infection Enzymes represented include superoxide dismutase (SOD), catalase (CAT), peroxidase (POD), and related antioxidant components as reported in the reviewed studies. Colour intensity reflects relative activity levels (green = upregulation; orange/red = downregulation), and arrows indicate direction and magnitude of change (↓ decreased, — no significant change, ↑ increased, ↑↑ strongly increased) relative to uninfected controls. Host categories are organised to illustrate graded antioxidant responses across susceptibility levels

*Oxidative stress and antioxidant enzymes* Upon nematode invasion, plants generate ROS such as hydrogen peroxide (H₂O₂), initiating defence signalling and localised cell death. In resistant genotypes, a rapid oxidative burst is followed by activation of ROS-scavenging enzymes like peroxidase, catalase, and SOD (Ji et al. 2015; Chavan et al. 2022). For instance, resistant pearl millet (*Pennisetum glaucum*) and rice lines showed significantly elevated enzyme activity, correlating with reduced galling and nematode fecundity (Singh et al. 2019, 2020). In contrast, susceptible cultivars across crops such as zucchini (*Cucurbita pepo*), mung bean, and tomato tend to exhibit delayed or insufficient antioxidant responses, leading to increased oxidative damage and nematode success (Abbasi & Hisamuddin 2014; Afifi et al. 2014). SSA-based studies reflect similar patterns, as seen in okra where tolerance was linked to moderate but timely enzymatic defence (Egedigwe et al. 2024). Relative activity changes in SOD, POD, CAT, and APX are shown in Fig. 3. Susceptible plants typically exhibit downregulation (↓) or no change (–), tolerant hosts show moderate upregulation (↑), and resistant plants display strong induction (↑↑), enabling early oxidative defence and nematode restriction. Data synthesised from multiple studies across different crops, including SSA and global contexts.

ROS responses require careful interpretation. A transient and spatially regulated oxidative burst is widely recognised as an early signalling event in plant defence, contributing to hypersensitive responses and downstream activation of defence pathways. In contrast, prolonged or excessive ROS accumulation may reflect oxidative stress and tissue damage, which can be associated with susceptibility rather than resistance. Several reviewed studies reported enhanced antioxidant enzyme activity in resistant genotypes, suggesting that effective resistance may depend not solely on ROS production, but on the capacity to regulate and detoxify ROS through coordinated antioxidant systems. Therefore, the functional relevance of ROS dynamics depends on timing, intensity, and redox balance rather than absolute accumulation levels.

*Phenolic compounds and secondary metabolites* Phenolic compounds, flavonoids, and other secondary metabolites function as chemical barriers against nematode invasion. Their upregulation has been consistently associated with resistance across several species, including tomato, cucumber, rice, and black nightshade (Rani et al. 2008; Nahar et al. 2011; Abed et al. 2023). In SSA studies, indigenous plants such as blackjack and dwarf aspilia (*Aspilia pluriseta*) demonstrated potent repellence and hatching inhibition mediated by volatile phenolics and sesquiterpenes (Mwamba et al. 2021; Kihika-Opanda et al. 2022). The phenolic pathway also feeds into lignin biosynthesis and cell wall reinforcement and is often activated in tandem with antioxidant enzyme production (Singh et al. 2019). Susceptible plants typically show downregulation or delayed accumulation of phenolics under infection pressure (Moslemi et al. 2016; Castaneda et al. 2017).

*Cell wall reinforcement* Effective defence often involves structural fortification of root tissues. The deposition of lignin, callose, and phenolic cross-links limits nematode mobility and disrupts feeding site formation. In resistant rice and tomato genotypes, increased expression of PAL and peroxidase coincides with early cell wall thickening (Medina et al. 2017; Singh et al. 2019). SSA studies have highlighted this mechanism in tolerant and resistant cultivars, with histochemical evidence of lignin deposition in cucumber and okra cultivars (Abed et al. 2023; Egedigwe et al. 2024). Conversely, susceptible cultivars lack this reinforcement, allowing gall development and giant cell formation (Niu et al. 2022). Although several defence mechanisms—such as ROS regulation, phenylpropanoid activation, and hormonal signalling—were recurrent across multiple crop species, caution is required when extrapolating findings from model or extensively studied crops to underutilised species. Anatomical features (e.g. root structure and vascular organisation), developmental dynamics, metabolic capacity, and genetic background may influence the magnitude and regulation of defence responses. Therefore, while general biochemical patterns provide useful conceptual frameworks, species-specific validation remains essential before translating resistance mechanisms across diverse cropping systems.

*Mechanistic integration with nematode feeding biology* These biochemical responses should be interpreted in the context of Meloidogyne feeding biology. Successful parasitism depends on the formation and maintenance of metabolically active giant cells that sustain nematode development. Regulated ROS production and phenylpropanoid activation may restrict feeding site establishment through localised cell death and cell wall reinforcement, whereas sustained oxidative imbalance may reflect susceptibility. Similarly, SA-, JA-, and ET-mediated signalling can influence defence activation, while successful infection often involves hormonal reprogramming that promotes giant cell formation. Thus, the functional significance of these pathways lies in their capacity to interfere with feeding site stability and nematode reproduction rather than acting as isolated biochemical events.

*Pathogenesis-related proteins* PR proteins such as PR-1, chitinases, and β−1,3-glucanases are classic outputs of SA- and JA-mediated signalling. Their upregulation is a reliable marker of resistance across species, particularly in *Mi-1*-gene-expressing tomato and induced rice lines (Branch et al. 2004; Medina et al. 2017; Chavan et al. 2022). Direct PR profiling has been reported less frequently in some regional studies; however, upstream hormone priming (e.g. with β-aminobutyric acid [BABA], dehydroascorbate [DHA], or chitosan oligosaccharide–oligogalacturonide [COS-OGA]) clearly activates downstream resistance genes, leading to effective nematode suppression (Ji et al. 2015; Singh et al. 2019).

*Hormonal crosstalk* The outcome of *Meloidogyne* infection is largely determined by hormonal balance. Resistance is often linked to early and strong SA and JA signalling, typically coupled with ET synergy (Nahar et al. 2011). Tolerant plants exhibit a more moderate hormonal response, sufficient to contain damage without triggering full hypersensitive resistance (Abed et al. 2023; Egedigwe et al. 2024). Susceptible hosts, however, show hormonal disruption, often characterised by nematode-induced elevation of auxin and ABA, which promote gall formation and suppress defence gene expression (Kyndt et al. 2016; Mbaluto et al. 2021). Brassinosteroid–JA antagonism also contributes to weakened resistance in some rice cultivars (Nahar et al. 2013). A distinction should be made between endogenous hormonal regulation triggered by Meloidogyne infection and exogenous hormone or elicitor treatments applied experimentally. While several studies reported intrinsic activation of SA-, JA-, or ET-associated pathways, others relied on external applications (e.g. BABA or SA analogues) to induce defence responses. These experimental treatments represent priming strategies rather than direct evidence of natural pathway activation.

While many studies reported increased ROS accumulation, antioxidant enzyme activity, phenolic content, and hormone-associated signalling following *Meloidogyne* infection, not all investigations established direct causal relationships between these biochemical changes and resistance outcomes. In several cases, elevated enzyme activity or metabolite accumulation was observed concurrently with reduced nematode reproduction; however, functional validation through gene silencing, inhibitor assays, transgenic approaches, or targeted pathway disruption was less frequently employed. Therefore, some reported defence markers may represent correlated stress responses rather than exclusively causal resistance mechanisms. Studies incorporating mechanistic validation, such as R-gene characterisation, hormone pathway manipulation, or controlled priming experiments, provide stronger evidence for functional involvement. Future research should prioritise experimental designs that distinguish defensive causality from secondary physiological responses.

### Comparative host response types

As illustrated in Fig. 2, the progression from susceptibility to resistance is marked by distinct shifts in oxidative, hormonal, and structural defences. For instance a study by Rani et al. (2008), reported that tomato susceptible cvs., ‘Pusa Ruby’ and ‘Pusa Early Dwarf’, recorded the lowest values for all the biochemical elements, whereas resistant cv., ‘PKM-1’, exhibited the greatest levels of POD and PPO enzyme activity as well as a high level of total phenols. Tolerance emerges as an intermediate state with partial biochemical activation and damage containment. Exposure of tomato cv. ‘Early Urbana’ to *M. javanica* resulted in moderate phenolic compounds, with reduced galling and nematode reproduction (Moslemi et al. 2016). Biochemically, tolerance may involve moderate defence activation, enhanced antioxidant regulation, or metabolic reallocation rather than strong hypersensitive responses. In contrast, partial resistance is typically characterised by measurable reductions in nematode reproduction or feeding site development. Because few reviewed studies applied standardised quantitative thresholds to define tolerance, interpretation was based on reported maintenance of plant growth parameters relative to infection severity within each experimental system. Tolerant genotypes allow some nematode entry and even limited gall formation but maintain growth through balanced antioxidant activity and partial hormone regulation (e.g. cucumber cv. ‘Babylon’) (Abed et al. 2023). In contrast, resistant plants exhibit early recognition, strong ROS bursts, structural fortification, and activation of SA/JA/PR pathways that collectively inhibit nematode establishment [e.g. rice treated with COS-OGA or DHA; (Singh et al. 2019; Chavan et al. 2022)]. Effective resistance is dependent on the timely and spatially coordinated activation of SA and JA/ET signalling, which exhibit antagonistic crosstalk.

The biochemical profiles of host plants reflect distinct defence strategies shaped by both genetic potential and environmental conditioning. In susceptible plants, defence pathways are suppressed, delayed, or hijacked, allowing nematodes to induce feeding sites and reproduce [e.g. tomato cv. ‘Money Maker’; (Murungi et al. 2018; Mbaluto et al. 2021)]. These profiles suggest a gradient of biochemical resilience, with tolerance emerging as a valuable, though under-researched, intermediary strategy, particularly relevant for crops in low-input farming systems in SSA. Murungi et al. (2018) identified three root volatiles (δ−3-carene, sabinene, and methyl salicylate) unique to tomato, whereas the other five (2-isopropyl-3-methoxypyrazine, 2-(methoxy)−3-(1-methylpropyl) pyrazine, tridecane, and α- and β-cedrene) were found in the root-emitted volatiles of both tomato and spinach. While 2-isopropyl-3-methoxypyrazine and tridecane enhanced the attraction of spinach, methyl salicylate significantly enhanced J2s attraction to tomato. Additionally, *M. incognita* J2s were more drawn to natural spinach root volatiles when methyl salicylate was added than to spinach volatiles by itself. This suggests that tomato volatiles’ methyl salicylate content significantly influences their preference for spinach.

## Implications for breeding and sustainable nematode management

The reviewed diverse biochemical defence mechanisms across different plants could significantly provide useful mechanistic insights that may support future resistance breeding methods and the sustainable control of *Meloidogyne* spp. in SSA and worldwide agricultural systems. Biochemical markers such as POD, PAL, and polyphenolic compounds have been often associated with elevated resistance phenotypes (Yu et al. 2022). By integrating the phenotypic characteristics of these markers into screening processes, breeders can advance the selection of resistant or tolerant cultivars. For example, yam landraces with greater POD and PAL activities also exhibited decreased galling and nematode reproduction, according to Abed et al. (2023), suggesting that these enzyme activities are reliable indicators of nematode resistance. Specifically, ET, SA, and JA phytohormone pathways play an important role in regulating gene expression during nematode invasion (Branch et al. 2004; Yang et al. 2018; Chavan et al. 2022). Plants can be prepared for faster responses to nematode infection and develop SAR by genetically or chemically altering these signalling networks. This may be particularly relevant for SSA since endogenous defence signalling may provide less costly alternatives to synthetic nematicides, although further field-based validation is necessary before practical deployment. The prevalence of laboratory and greenhouse-based studies across SSA highlights the critical need to validate biochemical resistance markers in field settings.

Given the complexity of soil ecosystems and interactions with microbial populations, the predominance of laboratory and greenhouse experiments limits direct extrapolation to field-scale nematode management. Nonetheless, biochemical resistance should be used in conjunction with agroecological approaches including crop rotation, intercropping, and organic amendments for sustainable nematode management (Coyne et al. 2018). Additionally, though not yet identified under *Meloidogyne*–host interactions, other secondary metabolites and enzymes unique to certain SSA crops have been discovered (Kubayi et al. 2025), indicating the possibility of bioprospecting local plants for nematodes that exist naturally. These compounds may reduce the need for hazardous synthetic poisons by functioning as bio-nematicides or as building blocks for environmentally beneficial insecticides. Breeding efforts must also include the quantitative and polygenic aspects of nematode resistance and combine biochemical phenotyping and genetic markers to capture complex defensive traits. Collaborative networks that integrate worldwide molecular biology resources with SSA research institutes may facilitate the use of genome editing and marker-assisted selection (MAS) techniques tailored for local germplasm. However, these applications remain largely prospective because most evidence currently derives from controlled experimental systems. Decisively, biochemical testing and resistance evaluation methods should be uniformly standardised throughout breeding programs for reliable comparison and data sharing. This harmonisation would facilitate the discovery of strong resistance traits and expedite cultivar development. Repurposing biochemical findings into practical breeding tools and integrated pest management techniques may contribute to more sustainable Meloidogyne management following adequate field validation. This is particularly beneficial for agricultural systems worldwide where nematode-susceptible staple crops form the foundation of food production.

While several reviewed studies highlight promising biochemical and molecular markers associated with resistance, the majority of available evidence derives from controlled laboratory or greenhouse experiments, with limited field-based validation, particularly in Sub-Saharan contexts. Therefore, recommendations related to marker-assisted selection, genome editing, and biochemical marker deployment should be interpreted as prospective and contingent upon further multi-environment testing and long-term field validation. Translational application requires careful evaluation of stability, heritability, and performance under diverse agronomic conditions.

## Knowledge gaps and research need in SSA and globally

*Limited research at the molecular omics level in SSA* SSA studies commonly employed spectrophotometric quantifications and enzymatic assays to quantify plant defence responses, alongside a notable use of GC–MS-based techniques for profiling volatile and secondary metabolites. Studies in this region seldom used molecular approaches such as transcriptomics, proteomics, or metabolomics. This contrasts sharply with international research carried out in countries such as Brazil and China (e.g. Castaneda et al. 2017; Sikandar et al. 2024), which extensively employed advanced molecular profiling to pinpoint gene regulatory networks and metabolic pathways. The absence of these methods in SSA restricts mechanistic insights and the discovery of novel resistance genes or biochemical markers, underscoring the critical need for capacity training and investment in state-of-the-art laboratory equipment.

*Underrepresentation of indigenous and underutilised crops* While most studies focus on commercial crops such as rice, potato and tomato, SSA studies have begun to examine local crops including black nightshade, blackjack, and Asteraceae species (Mwamba et al. 2021; Kihika-Opanda et al. 2022). However, research on underutilised indigenous plants is still at its infancy (Mmbando 2025). These species should be prioritised in future research, integrating field-based, molecular, and biochemical assessments, due to their importance for agro-biodiversity and regional food security.

*Inadequate field-based and longitudinal research* Most research, especially in SSA, used artificial inoculants and was conducted in laboratory or greenhouse environments (Kihika et al. 2017; Mwamba et al. 2021). Few studies assessed biochemical defence responses in outdoor settings or throughout many developmental stages (Kihika-Opanda et al. 2022; Egedigwe et al. 2024). Our understanding of how soil microbiomes, environmental conditions, and agricultural practices impact plant–nematode interactions in real-world settings is hampered by this information gap.

*Limited study of hormonal crosstalk and signalling pathways* Despite being acknowledged as essential to plant defence (Kyndt et al. 2016; Sikder et al. 2021), the dynamic interactions and crosstalk of the ET, SA, and JA pathways are still poorly understood, especially in SSA crops. Global studies have demonstrated complex regulation networks and are increasingly using hormonal profiling and gene expression analysis. By combining these techniques, SSA researchers might identify key hormone regulators that could be the target of breeding or biocontrol initiatives.

It is important to interpret these regional patterns with caution. Differences in analytical approaches, methodological depth, and research representation may partly reflect disparities in research infrastructure, funding priorities, access to advanced analytical platforms, and publication visibility rather than intrinsic biological variation in plant defence mechanisms. Database indexing practices and language-related publication biases may further influence study retrieval and representation. Consequently, the apparent dominance of specific defence pathways or methodological approaches in certain regions should be understood within the broader context of structural research environments. Therefore, conclusions regarding sustainable management implications should be interpreted cautiously until supported by broader field-based evidence.

## Review limitations and future directions

This comprehensive review offers important insights into plant biochemical defence systems against *Meloidogyne* spp., despite several constraints to consider. The sole inclusion of peer-reviewed English-language studies may have inadvertently excluded pertinent material published in other languages or in grey literature. For research conducted outside of SSA, this is especially crucial. Variations in experimental designs, biochemical assays, and reporting rules between studies hindered direct comparison and comprehensive meta-analysis. The use of biochemical markers, nematode inoculum density, sampling intervals following infection, and variations in plant developmental stages all restrict the generalisability of certain results and emphasise the need for standardised protocols. The data’s applicability to field settings, where complex interactions with soil microbiota, changing abiotic factors, and management strategies impact plant–nematode dynamics, was restricted by the fact that most studies were conducted in controlled greenhouse or laboratory environments. A greater emphasis on field-based and longitudinal research would enhance the ecological importance and use of biochemical resistance markers. Despite efforts to incorporate SSA perspectives, a more complete mechanistic understanding of resistance in local crops is limited by the absence of high-resolution molecular and omics-level data from the region. Building the capacity and infrastructure to enable advanced molecular research in SSA is therefore essential.

Additionally, by focussing on plant biochemical responses, this study did not investigate interactions involving microbial symbionts, nematode-associated bacteria, or co-occurring pathogens that can change infection outcomes. Expanding the scope of nematode resistance research to include these multitrophic interactions will make it more thorough. Limited research has also been done on the connection between nematode infection and plant defence mechanisms and abiotic and biotic stressor combinations such as drought, malnutrition, or severe temperatures. To develop crop varieties and management strategies that are climate-adaptable, it is important to examine these linkages. To further develop this domain in subsequent years, multidisciplinary research in many environmental contexts must incorporate molecular, biochemical, ecological, and agronomic approaches. Standardised experimental protocols and the creation of biochemical marker panels will improve comparability and reproducibility. Detailed molecular characterisation and field validation are necessary to prioritise native and underutilised crops in SSA. Emerging technologies including transcriptomics, metabolomics, gene editing, and high-throughput phenotyping offer promising methods for identifying and managing significant resistance mechanisms. Additionally, by including climate change scenarios into research frameworks, it will ensure the development of strong and sustainable nematode management plans. By embracing these research avenues and overcoming these obstacles, the scientific community may enhance sustainable management of *Meloidogyne* spp. and boost food security, particularly in vulnerable SSA agroecosystems.

## Conclusion

This review confirms that *Meloidogyne* spp. are the most prolific soilborne pests as evidenced by the current review attesting to relatively multiple agricultural crops that are susceptible. This thorough research elucidated key biochemical defence mechanisms that plants employ against *Meloidogyne* spp. Common responses were noted, such as the participation of phytohormone signalling pathways like ET, SA, and JA, the accumulation of phenolic compounds, and enhanced activity of enzymes such POD and PAL. In a range of crops, these biochemical markers were shown to be strongly correlated with host resistance and tolerance. Only a small number of field-based and molecular investigations were conducted by SSA, which mostly employed controlled enzymatic assays. Global research is increasingly using contemporary omics technology to unravel intricate resistance networks, even if study scope and methodological rigour vary significantly by region. Because most included studies were conducted under controlled laboratory or greenhouse conditions, further multi-environment and field-based validation is required before translating these biochemical findings into large-scale nematode management recommendations. The underrepresentation of native crops and field validations in SSA highlights important information gaps that need to be addressed. Increasing the capacity for molecular research and standardising biochemical testing in SSA are essential to transforming these findings into practical treatments. Biochemical resistance markers may support future breeding programmes and integrated nematode management strategies, while resistant cultivars combined with sound agronomic practices remain central to sustainable *Meloidogyne* management. To successfully combat *Meloidogyne* spp., especially in SSA, policymakers must prioritise nematology research infrastructure and capacity growth. Innovative technologies and processes may be implemented more quickly by promoting technology transfer and interdisciplinary collaboration. Additionally, based on biochemical knowledge, agricultural extension organisations ought to promote the application of sustainable, locally specific nematode control strategies.

## Data Availability

The data that support the findings of this study are available on request from the corresponding author.

## References

[CR1] Abbasi P, Hisamuddin N (2014) Effect of different inoculum levels of *Meloidogyne incognita* on growth and biochemical parameters of *Vigna radiata*. Asian J Nematol 3:15–20. 10.3923/ajn.2014.15.20

[CR2] Abdulsalam S, Peng H, Yao Y, Fan L, Jiang R, Shao H, Zhang Y, Huang W, Kong LA, Peng D (2021) Prevalence and molecular diversity of plant-parasitic nematodes of yam (*Dioscorea* spp.) in China, with focus on *Merlinius* spp. Biol 10:1299. 10.3390/biology10121299 10.3390/biology10121299 PMC8699026 34943214

[CR3] Abed A, Fayyad M, Heidari R (2023) Study of some biochemical changes of some cultivars of cucumber when infected with the root-knot nematode *Meloidogyne* sp in southern Iraq. Bionatura 8:1–8. 10.21931/RB/CSS/2023.08.02.40

[CR4] Afifi A, Al-Sayed A, Mahfoud N, Farahat A (2014) Enzymatic and non-enzymatic oxidants and antioxidants involved in defense mechanisms against root-knot, reniform and citrus nematodes in their hosts. Egypt J Agronematol 13:172–188. 10.21608/ejaj.2014.63693

[CR5] Banora MY, Almaghrabi OA (2019) Differential response of some nematode-resistant and susceptible tomato genotypes to *Meloidogyne javanica* infection. J Plant Prot Res 59:113–123. 10.24425/jppr.2019.126040

[CR6] Bernard GC, Khan MR (2025) Understanding the mechanisms of nematode disease complexes. In: Khan N (ed) Nematode disease complexes in agricultural crops. CABI, Wallingford, pp 47–66

[CR7] Bogale M, Baniya A, DiGennaro P (2020) Nematode identification techniques and recent advances. Plants (Basel) 24:1260. 10.3390/plants9101260 10.3390/plants9101260 PMC7598616 32987762

[CR8] Bowman A, Chisoro S (2024) Inclusive agro-industrial development and sectoral systems of innovation: insights from South Africa. Innov Dev 15:285–314. 10.1080/2157930X.2024.2312311

[CR9] Branch C, Hwang CF, Navarre DA, Williamson VM (2004) Salicylic acid is part of the Mi-1-mediated defense response to root-knot nematode in tomato. Mol Plant Microbe Interact 17:351–356. 10.1094/mpmi.2004.17.4.351 15077667 10.1094/MPMI.2004.17.4.351

[CR10] Castaneda NEN, Alves GSC, Almeida RM, Amorim EP, Fortes Ferreira C, Togawa RC, Costa MMDC, Grynberg P, Santos JRP, Cares JE (2017) Gene expression analysis in *Musa acuminata* during compatible interactions with *Meloidogyne incognita*. Ann Bot 119:915–930. 10.1093/aob/mcw272 28130221 10.1093/aob/mcw272 PMC5604581

[CR11] Chavan SN, De Kesel J, Desmedt W, Degroote E, Singh RR, Nguyen GT, Demeestere K, De Meyer T, Kyndt T (2022) Dehydroascorbate induces plant resistance in rice against root-knot nematode *Meloidogyne graminicola*. Mol Plant Pathol 23:1303–1319. 10.1111/mpp.13230 35587614 10.1111/mpp.13230 PMC9366072

[CR12] Coyne DL, Cortada L, Dalzell JJ, Claudius-Cole AO, Haukeland S, Luambano N, Talwana H (2018) Plant-parasitic nematodes and food security in Sub-Saharan Africa. Annu Rev Phytopathol 56:381–403. 10.1146/annurev-phyto-080417-045833 29958072 10.1146/annurev-phyto-080417-045833 PMC7340484

[CR13] Danish M, Robab MI, Marraiki N, Shahid M, Zaghloul NS, Nishat Y, Shaikh H (2021) Root-knot nematode *Meloidogyne incognita* induced changes in morpho-anatomy and antioxidant enzymes activities in *Trachyspermum ammi* (L.) plant: A microscopic observation. Physiol Mol Plant Pathol 116:101725. 10.1016/j.pmpp.2021.101725

[CR14] dos Santos MF, da Silva Mattos V, Monteiro JM, Almeida MR, Jorge AS Jr, Cares JE, Castagnone-Sereno P, Coyne D, Carneiro RM (2019) Diversity of *Meloidogyne* spp. from peri-urban areas of Sub-Saharan Africa and their genetic similarity with populations from the Latin America. Physiol Mol Plant Pathol 105:110–118. 10.1016/j.pmpp.2018.08.004

[CR15] Egedigwe U, Udengwu O, Ekeleme-Egedigwe C, Maduakor C, Urama C, Odo C, Ojua E (2024) Integrated stress responses in okra plants (cv.Meya’): Unravelling the mechanisms underlying drought and nematode co-occurrence. BMC Plant Biol 24:986. 10.1186/s12870-024-05686-1 39427110 10.1186/s12870-024-05686-1 PMC11490165

[CR16] Eisenback JD, Triantaphyllou HH (2020) Root-knot nematodes: *Meloidogyne* species and races. In: Wickle WR (ed) Manual of agricultural nematology. CRC Press, New York, pp 191–274

[CR17] Goellner M, Wang X, Davis EL (2001) Endo-β-1, 4-glucanase expression in compatible plant–nematode interactions. Plant Cell 13:2241–2255. 10.1105/tpc.010219 11595799 10.1105/tpc.010219 PMC139156

[CR18] Habiyaremye A, Ncube P, Sichoongwe K, Slater A (2024) The use of advanced technology in South African agriculture: insights from selected sub-sectors. SARChI Industrial Development Working Paper Series, DSI/NRF South African research chair in industrial development

[CR19] Ji H, Kyndt T, He W, Vanholme B, Gheysen G (2015) β-Aminobutyric acid–induced resistance against root-knot nematodes in rice is based on increased basal defense. Mol Plant Microbe Interact 28:519–533. 10.1094/mpmi-09-14-0260-r 25608179 10.1094/MPMI-09-14-0260-R

[CR20] Kantor C, Eisenback JD, Kantor M (2024) Biosecurity risks to human food supply associated with plant-parasitic nematodes. Front Plant Sci. 10.3389/fpls.2024.1404335 38745921 10.3389/fpls.2024.1404335 PMC11091314

[CR21] Karuri H (2022) Root and soil health management approaches for control of plant-parasitic nematodes in sub-Saharan Africa. Crop Prot 152:105841. 10.1016/j.cropro.2021.105841

[CR22] Khan A, Ansari SA, Haris M, Hussain T, Khan AA (2023) *Meloidogynespecies*: threat to vegetable produce. In: Ahmad F, Blázquez GN (eds) Root-galling disease of vegetable plants. Springer, Singapore, pp 61–83

[CR23] Kihika R, Murungi LK, Coyne D, Ng’ang’a M, Hassanali A, Teal PEA, Torto B (2017) Parasitic nematode *Meloidogyne incognita* interactions with different *Capsicum annum* cultivars reveal the chemical constituents modulating root herbivory. Sci Rep 7:2903. 10.1038/s41598-017-02379-8 28588235 10.1038/s41598-017-02379-8 PMC5460232

[CR24] Kihika-Opanda R, Tchouassi DP, Ng’ang’a MM, Beck JJ, Torto B (2022) Chemo-ecological insights into the use of the non-host plant vegetable black-jack to protect two susceptible solanaceous crops from root-knot nematode parasitism. J Agric Food Chem 70:6658–6669. 10.1021/acs.jafc.2c01748 35613461 10.1021/acs.jafc.2c01748

[CR25] Kubayi S, Makola RT, Dithebe K (2025) Exploring the antimicrobial, antioxidant and extracellular enzymatic activities of culturable endophytic fungi isolated from the leaves of *Kirkia acuminata* Oliv. Microorganisms 13:692. 10.3390/microorganisms13030692 40142584 10.3390/microorganisms13030692 PMC11945046

[CR26] Kyndt T, Goverse A, Haegeman A, Warmerdam S, Wanjau C, Jahani M, Engler G, de Almeida Engler J, Gheysen G (2016) Redirection of auxin flow in *Arabidopsis thaliana* roots after infection by root-knot nematodes. J Exp Bot 67:4559–4570. 10.1093/jxb/erw230 27312670 10.1093/jxb/erw230 PMC4973730

[CR27] Mbaluto CM, Vergara F, van Dam NM, Martínez-Medina A (2021) Root infection by the nematode *Meloidogyne incognita* modulates leaf antiherbivore defenses and plant resistance to *Spodoptera exigua*. J Exp Bot 72:7909–7926. 10.1093/jxb/erab370 34545935 10.1093/jxb/erab370 PMC8664589

[CR28] Medina IL, Gomes CB, Correa VR, Mattos VS, Castagnone-Sereno P, Carneiro RM (2017) Genetic diversity of *Meloidogyne* spp. parasitising potato in Brazil and aggressiveness of *M. javanica* populations on susceptible cultivars. Nematol 19:69–80. 10.1163/15685411-00003032

[CR29] Mmbando GS (2025) The current challenges and solutions on utilizing the potential of underutilized and neglected crops for Africa’s food security. Discov Plants 2:163. 10.1007/s44372-025-00261-w

[CR30] Moslemi F, Fatemy S, Bernard F (2016) Inhibitory effects of salicylic acid on *Meloidogyne javanica* reproduction in tomato plants. Span J Agric Res 14:e1001–e1001. 10.5424/sjar/2016141-8706

[CR31] Murungi LK, Kirwa H, Coyne D, Teal PE, Beck JJ, Torto B (2018) Identification of key root volatiles signaling preference of tomato over spinach by the root knot nematode *Meloidogyne incognita*. J Agric Food Chem 66:7328–7336. 10.1021/acs.jafc.8b03257 29938509 10.1021/acs.jafc.8b03257

[CR32] Mwamba S, Kihika-Opanda R, Murungi LK, Losenge T, Beck JJ, Torto B (2021) Identification of repellents from four non-host Asteraceae plants for the root knot nematode, *Meloidogyne incognita*. J Agric Food Chem 69:15145–15156. 10.1021/acs.jafc.1c06500 34882384 10.1021/acs.jafc.1c06500

[CR33] Nahar K, Kyndt T, De Vleesschauwer D, Höfte M, Gheysen G (2011) The jasmonate pathway is a key player in systemically induced defense against root knot nematodes in rice. Plant Physiol 157:305–316. 10.1104/pp.111.177576 21715672 10.1104/pp.111.177576 PMC3165880

[CR34] Nahar K, Kyndt T, Hause B, Höfte M, Gheysen G (2013) Brassinosteroids suppress rice defense against root-knot nematodes through antagonism with the jasmonate pathway. Mol Plant Microbe Interact 26:106–115. 10.1094/mpmi-05-12-0108-fi 23194343 10.1094/MPMI-05-12-0108-FI

[CR35] Nature Index (2016) 2017 Research leaders: Leading institutions. https://www.nature.com/nature-index/research-leaders/2017/institution/all/all/countries-Kenya. Accessed 07 May 2025

[CR36] Niu Y, Xiao L, de Almeida-Engler J, Gheysen G, Peng D, Xiao X, Huang W, Wang G, Xiao Y (2022) Morphological characterisation reveals new insights into giant cell development of *Meloidogyne graminicola* on rice. Planta 255:70. 10.1007/s00425-022-03852-z 35184234 10.1007/s00425-022-03852-z PMC8858295

[CR37] Onkendi EM, Kariuki GM, Marais M, Moleleki LN (2014) The threat of root-knot nematodes (*Meloidogyne* spp.) in Africa: A review. Plant Pathol 63:727–737. 10.1111/ppa.12202

[CR38] Page MJ, McKenzie JE, Bossuyt PM, Boutron I, Hoffmann TC, Mulrow CD, Shamseer L, Tetzlaff JM, Akl EA, Brennan SE, Chou R, Glanville J, Grimshaw JM, Hróbjartsson A, Lalu MM, Li T, Loder EW, Mayo-Wilson E, McDonald S, McGuinness LA, Stewart LA, Thomas J, Tricco AC, Welch VA, Whiting P, Moher D (2021) The PRISMA 2020 statement: An updated guideline for reporting systematic reviews. BMJ. 10.1136/bmj.n71 33782057 10.1136/bmj.n71 PMC8005924

[CR39] Przybylska A, Kornobis F, Obrępalska-Stęplowska A (2018) Analysis of defense gene expression changes in susceptible and tolerant cultivars of maize (*Zea mays*) upon *Meloidogyne arenaria* infection. Physiol Mol Plant Pathol 103:78–83. 10.1016/j.pmpp.2018.05.005

[CR40] Qi J, Li J, Han X, Li R, Wu J, Yu H, Hu L, Xiao Y, Lu J, Lou L (2016) Jasmonic acid carboxyl methyltransferase regulates development and herbivory-induced defense response in rice. J Integr Plant Biol 58:564–576. 10.1111/jipb.12436 26466818 10.1111/jipb.12436

[CR41] Ramatsitsi N, Ramachela K (2023) Histological characterisation of wild cucumber resistance to *Meloidogyne* species. J Plant Dis Prot 130:883–889. 10.1007/s41348-023-00733-9

[CR42] Ramatsitsi MN, Nxumalo S, Ramachela K, Khosa MC (2024) Host response of five potato cultivars to *Meloidogyne* nematodes. J Plant Dis Prot 131:891–898. 10.1007/s41348-023-00851-4

[CR43] Rani CI, Veeraragavathatham D, Sanjutha S (2008) Analysis on biochemical basis of root knot nematode (*Meloidogyne incognita*) resistance in tomato (*Lycopersicon esculentum* Mill.). Res J Agric Biol Sci 4:866–870

[CR44] Santos MD, Curtis R, Abrantes I (2013) Effect of plant elicitors on the reproduction of the root-knot nematode *Meloidogyne chitwoodi* on susceptible hosts. Eur J Plant Pathol 136:193–202. 10.1007/s10658-012-0155-6

[CR45] Sikandar A, Wu F, He H, Ullah RMK, Wu H (2024) Growth, physiological, and biochemical variations in tomatoes after infection with different density levels of *Meloidogyne enterolobii*. Plants 13:293 38256846 10.3390/plants13020293 PMC10819788

[CR46] Sikder MM, Vestergård M, Kyndt T, Kudjordjie EN, Nicolaisen M (2021) Phytohormones selectively affect plant parasitic nematodes associated with *Arabidopsis* roots. New Phytol 232:1272–1285. 10.1111/nph.17549 34115415 10.1111/nph.17549

[CR47] Singh RR, Chinnasri B, De Smet L, Haeck A, Demeestere K, Van Cutsem P, Van Aubel G, Gheysen G, Kyndt T (2019) Systemic defense activation by COS-OGA in rice against root-knot nematodes depends on stimulation of the phenylpropanoid pathway. Plant Physiol Biochem 142:202–210. 10.1016/j.plaphy.2019.07.003 31302409 10.1016/j.plaphy.2019.07.003

[CR48] Singh G, Kanwar RS, Sharma L, Neeraj Chugh LK, Kaushik P (2020) Biochemical changes induced by *Meloidogyne graminicola* in resistant and susceptible pearl millet (*Pennisetum glaucum* L.) hybrids. Plant Pathol J 19:132–139. 10.3923/ppj.2020.132.139

[CR49] Tiwari S (2024) Impact of nematicides on plant-parasitic nematodes: challenges and environmental safety. Tunis J Plant Prot 19:101–120. 10.4314/tjpp.v19i2.4

[CR50] Wong CKF, Teh CY (2021) Impact of biofertilizers on horticultural crops. In: Inamuddin, Ahamed MI, Boddula R, Rezakazemi M (eds) Biofertilizers: study and impact. John Wiley & Sons, New Jersey, pp 39–103

[CR51] Xie X, Lu J, Lin R, Ling J, Mao Z, Zhao J, Yang Q, Zheng S, Li Y, Visser RGF, Bai Y, Xie B (2025) Comparative transcriptomics of susceptible and resistant *Cucumis metuliferus* upon *Meloidogyne incognita* infection. Planta 261:72. 10.1007/s00425-025-04649-6 40029419 10.1007/s00425-025-04649-6

[CR52] Yang YX, Wu C, Ahammed GJ, Wu C, Yang Z, Wan C, Chen J (2018) Red light-induced systemic resistance against root-knot nematode is mediated by a coordinated regulation of salicylic acid, jasmonic acid and redox signaling in watermelon. Front Plant Sci 9:899. 10.3389/fpls.2018.00899 30042771 10.3389/fpls.2018.00899 PMC6048386

[CR53] Yu J, Yu X, Li C, Ayaz M, Abdulsalam S, Peng D, Qi R, Peng H, Kong L, Jia J, Huang W (2022) Silicon mediated plant immunity against nematodes: Summarising the underline defence mechanisms in plant nematodes interaction. Int J Mol Sci 23:14026. 10.3390/ijms232214026 36430503 10.3390/ijms232214026 PMC9692242

[CR54] Zhou W, Arcot Y, Medina RF, Bernal J, Cisneros-Zevallos L, Akbulut ME (2024) Integrated pest management: An update on the sustainability approach to crop protection. ACS Omega 9:41130–41147. 10.1021/acsomega.4c06628 39398119 10.1021/acsomega.4c06628 PMC11465254

